# Pericapsular nerve group block followed by regional anesthesia for pathological fracture fixation in a multiple myeloma patient

**DOI:** 10.1002/ccr3.9374

**Published:** 2024-08-29

**Authors:** Aayusha Suwal, Nischal Subedi, Nischal Shrestha, Ujma Shrestha

**Affiliations:** ^1^ Kathmandu Medical College and Teaching Hospital Kathmandu Nepal; ^2^ Department of Anesthesiology Kathmandu Medical College and Teaching Hospital Kathmandu Nepal

**Keywords:** anesthetic complications, multiple myeloma, pericapsular nerve group block, regional anesthesia

## Abstract

Appropriate anesthetic technique for fracture fixation in multiple myeloma is crucial in minimizing perioperative complications. The use of pericapsular nerve group block followed by spinal anesthesia for the operative management of pathological fracture in multiple myeloma patient along with proper pre‐ and postanesthetic care ensures better outcome for the patient.

**Abstract:**

Multiple myeloma is a malignant plasma cell disease that often presents with anemia, renal failure, hyperkalemia and osteolytic bone lesions. The advancements in drug therapy of multiple myeloma have prolonged the lifespan of the affected people, resulting in a rise in cases of surgical management of fractures in such patients. Anesthetic management, despite being of utmost importance in minimizing perioperative complications in such patients, has not been widely studied, especially in this part of the world. Hence, we report a case of 64 years diabetic, HbSAg positive male with hypothyroidism and a known case of multiple myeloma since the last 6 years, under medication for his comorbidities who suffered acetabular fracture. In this case report, the use of pericapsular nerve group block followed by spinal anesthesia for the operative management of the fracture has been discussed along with several pre‐ and postanesthetic considerations. With appropriate anesthetic techniques and proper pre‐ and postanesthetic care, better outcomes can be guaranteed.

## 1 INTRODUCTION

Multiple myeloma is a malignant plasma cell disease that accounts for 10% of all hematologic malignancies.^1^, ^2^ Complications associated with this condition often include anemia, renal failure, hypercalcemia, recurrent infections, and osteolytic bone lesions which may present with bone pain and easy fractures.^3^, ^4^ Because of the various risks of multiple myeloma, it is imperative for anesthesiologists to maintain an uneventful preoperative, intraoperative, and postoperative period for any multiple myeloma patient undergoing surgery. Advances in modern multiple myeloma treatment have increased the life expectancy of patients and also increased the requirement of surgical interventions for complications like fracture. Although fractures are common in patients with multiple myeloma, the administration of anesthetics in its management has not been widely studied, so this case study discusses the anesthetic management in such a patient to minimize complications from both the disease as well as the anesthetic components. Pericapsular nerve group (PENG) block with relatively low impact on hemodynamics is an ideal analgesia for pain‐free positioning of the patient for spinal anesthesia.^5^, ^6^ Spinal anesthesia has lesser complications than general anesthesia in the already delicate case of multiple myeloma including lower risk of thromboembolism, respiratory depression, myocardial infarct, renal failure, transfusion requirement, and ICU admission.^7^, ^8^, ^9^, ^10^ The use of PENG block followed by spinal anesthesia along with various pre‐ and postanesthetic considerations has been discussed here.

## 2 CASE HISTORY AND EXAMINATION

**FIGURE 1:**
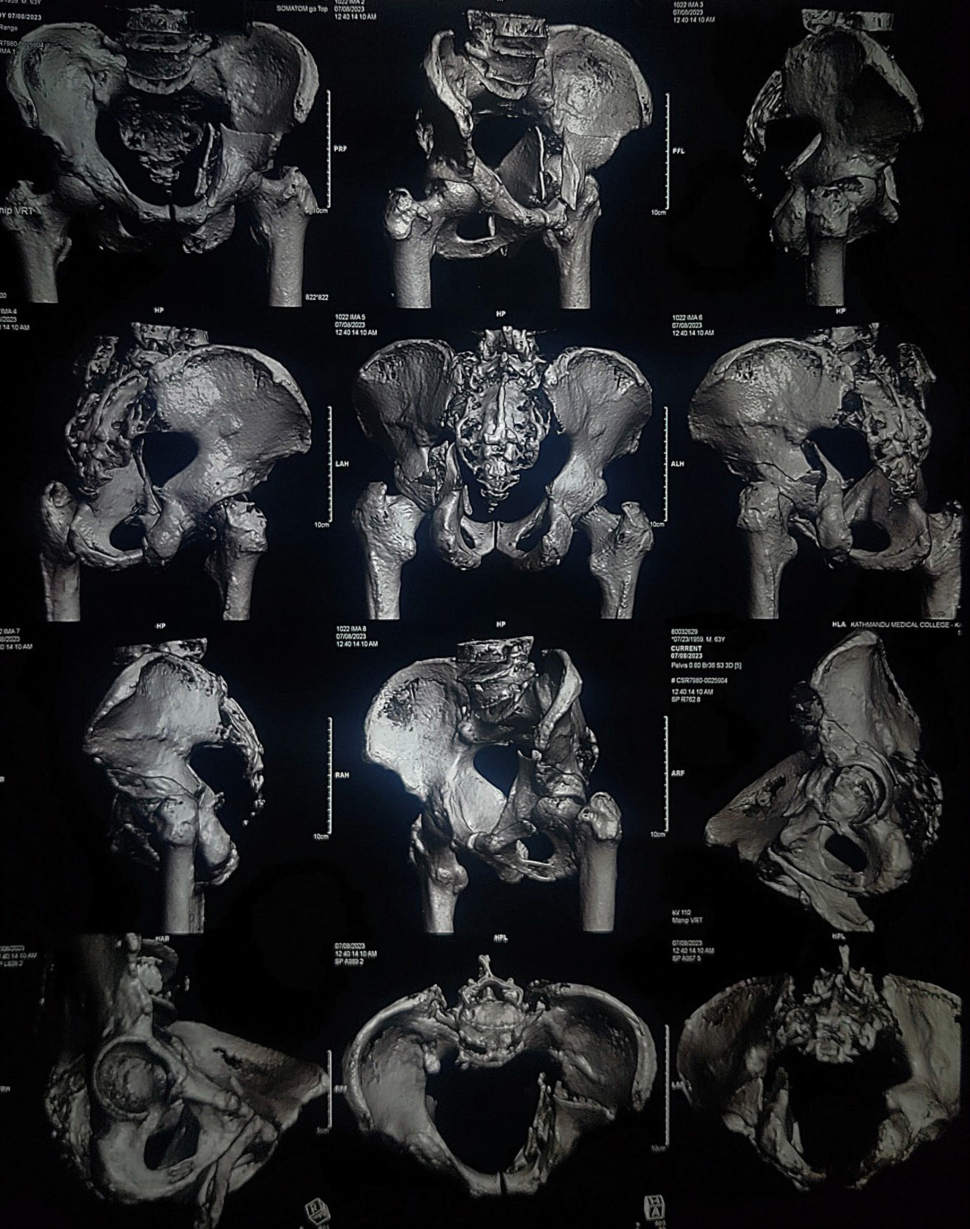
Nonenhanced computed tomography (NECT) of pelvis with 3D reconstruction.

A 64 years diabetic HbSAg positive male with hypothyroidism weighing 70 kg presented to emergency with complaints of pain over the left hip for 2 days following an alleged history of slipping on the floor of the kitchen. The patient was a known case of multiple myeloma, diagnosed 6 years back, with history of two bone marrow transplantations since the diagnosis, the last one being 8 months prior to the time of presentation. He had taken intravenous Bortezumib 2.5 mg once a week for 4 months after the diagnosis and had previously undergone numerous blood transfusions due to low hemoglobin. Before the surgery, he had been taking oral doses of lenalidomide 10 mg once a day for 21 days per month. He had also been taking entecavir every day since the time when his blood tests came back positive for HbSAg 5 years ago. He had also been taking oral levothyroxine 25 mcg since his diagnosis of hypothyroidism 3 years prior. He was also under oral hypoglycemics namely metformin 1000 mg and voglibose 0.3 mg twice a day for diabetes mellitus. Following his hospital visit, Nonenhanced computed tomography (NECT) of pelvis with 3D reconstruction was done which revealed anterior column with posterior hemitransverse left acetabular fracture along with multiple variable sized lytic lesions with sclerotic and irregular margin in bilateral iliac bones, visualized section of bilateral proximal femur, L4, L5 and sacral vertebrae as shown in Figure 1. An open reduction and internal fixation was planned for which preanesthetic assessment was done.

On examination conducted as a part of his preanesthetic assessment, his vitals were stable and systemic examinations showed normal findings. Local examination revealed swelling and tenderness over the left hip. Airway examination showed short neck and Mallampati grading of II.

## 3 METHODS

The laboratory investigations of the patient showed decreased hemoglobin of 9.4 g/dL (normal: 12 g/dL–16 g/dL), decreased total white blood cell count of 4900/mm^3^ (normal: 4000/mm^3^–11,000/mm^3^) and a platelet count of 150,000/mm^3^ (normal: 150,000/mm^3^–450,000/mm^3^). His urea, creatinine and sodium levels were within normal limits but he had a low serum potassium level of 3.6 mEq/L (normal: 3.8 mEq/L–5 mEq/L). His PT/INR and liver function tests were also within the normal range. His chest X‐ray revealed hyperinflated bronchovascular margins, electrocardiography showed normal sinus rhythm and echocardiography revealed normal heart chambers with left ventricular ejection fraction of 60%. Preliminary diagnosis of American Society of Anesthesiologist (ASA) Physical Status Grade III with bicolumnar fracture of left acetabulum with multiple myeloma, diabetes mellitus and hypothyroidism with HbSAg positive status and probable difficult airway was made.

**FIGURE 2:**
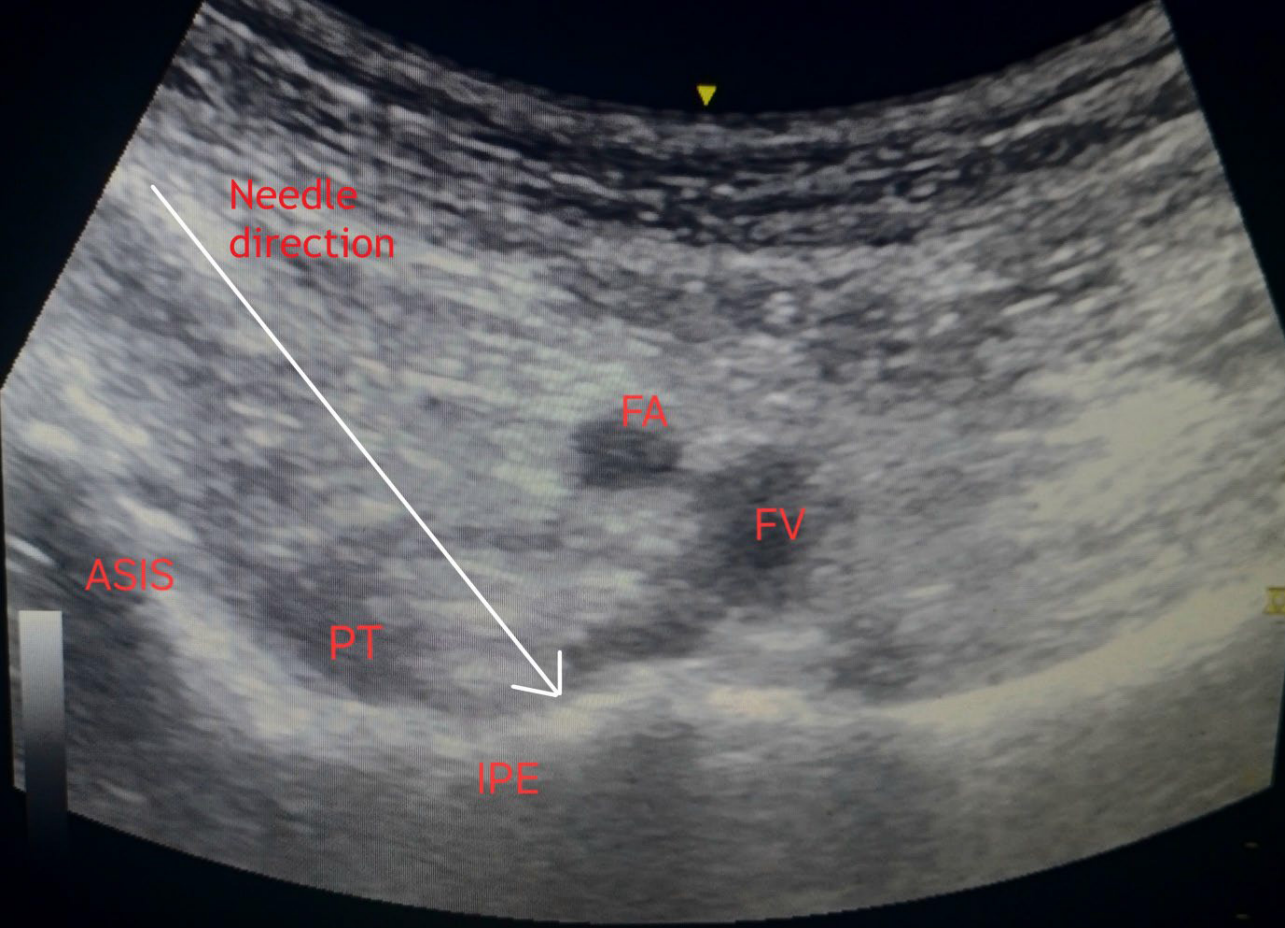
Pericapsular nerve group (PENG) block. ASIS, anterior superior iliac spine; FA, femoral artery; FV, femoral vein; IPE, iliopubic eminence; PT, psoas tendon.

In the operating room, with informed consent, PENG block was given to obliterate pain during positioning for spinal anesthesia. In supine position following removal of traction and sedation with bolus intravenous injection of fentanyl 50 mcg, preliminary scan was done by placing the low frequency curvilinear ultrasound probe in a transverse plane over the anterior inferior iliac spine and rotating it 45 degrees counter clockwise to align with the pubic ramus. The iliopubic eminence, the iliopsoas muscle tendon, the femoral artery and pectineus muscle were located and femoral nerve was identified. Under asepsis, ultrasound compatible 25G Quincke spinal needle was inserted from lateral to medial in an in‐plane approach, with the aim of placing the tip in the musculofascial plane between the psoas tendon anteriorly and the pubic ramus posteriorly as shown in Figure 2, with total 20 mL local anesthetic (two 10 mL syringes each containing 5 mL of 2% lignocaine with adrenaline (2:  100000) and 5 mL of 0.25% bupivacaine plain after dilution). At the time of injection and spread of local anesthetics, caution was taken to visualize the tip of the needle and frequent negative aspiration done.

Then the patient was positioned right laterally, and under aseptic conditions, L1‐L2 space was identified because of degenerative changes in the vertebra of the patient below L2 level. Through midline approach, 25 G Quincke needle was inserted into the subarachnoid space and 3.4 mL 0.5% heavy bupivacaine with 0.1 mL morphine and 0.1 mL dexmedetomidine was delivered. Anesthetic level of T12 was achieved initially and final level achieved was T6.

In the 4 h of intraoperative period, his blood pressure ranged from 87 to 120 mm of Hg of systolic and 53–70 mm of Hg of diastolic blood pressure. He received inj. Mephentermine bolus at two instances (9 mg and 3 mg each) for low Mean Arterial Pressure (MAP <65 mm of Hg). Oxygen saturation was maintained throughout at 99% via face mask at 5 L/h. His urine output was maintained at 2.3 mL/kg/h. His estimated blood loss intraoperatively was 400 mL.

In the postoperative ward, pain was managed using dexmedetomidine infusion at 4.5 mL/h and paracetamol 1 g intravenous infusion every 6 h. Supplemental oxygen continued for 16 h postoperatively. Enteral feeding was started 6 h after the surgery and mobilization began 8 days after the surgery. For myelosuppressive state of the patient, whole blood transfusion was done on second, third and fifth postoperative day. Lasix was started on the second postoperative day for decreased urine output and a Foley's catheter was placed to overcome urinary retention. Regular insulin was started on a sliding scale from the third postoperative day and incentive spirometry was started from the fourth postoperative day. Linalidomide was resumed 10 days after it was halted for surgery. There were no wound infections and renal function was optimal and the patient was discharged 10 days after the surgery.

## 4 DISCUSSION

Multiple myeloma is a malignant plasma cell disorder that accounts for 1% of all malignancies and about 10% of all hematological malignancies, with a median age of diagnosis of 66 years.^1^, ^2^ International Myeloma Working Group consensus defines multiple myeloma as a malignancy with 10% or more clonal bone marrow plasma cells or biopsy‐proven bony or extramedullary plasmacytoma along with one or more of myeloma defining events like hypercalcemia, renal failure, anemia, and osteolytic bone lesions.^3^ Multiple myeloma can present with fatigue, bone pain, easy bruising and bleeding, recurrent infections as a result of underlying anemia, hypercalcemia, lytic bone lesions, thrombocytopenia and hypogammaglobulinemia.^4^ Conventional radiography of nearly 75% of MM patients is known to show punched‐out lytic lesions, osteoporosis or fractures, and nearly two‐thirds of patients present with bone pain, which might be of sudden onset with pathological fractures.^2^ While infections constitute majority of myeloma‐related deaths, renal failure is seen in one‐fifth of MM patients, of which 10% need dialysis.^11^ Venous thromboembolism is another common complication seen in MM.^12^ Multiple myeloma patients were more likely to experience a number of postoperative side effects, including acute renal failure, surgical site infection, sepsis, and in‐hospital pneumonia.^13^

Newer treatments for MM have increased the median survival years for multiple myeloma, which has also increased the requirement of surgical interventions for some complications like fracture. Since MM can lead to a variety of complications, anesthesiologists must take utmost precautions to ensure that the perioperative period remains uneventful during any surgical intervention in a patient with multiple myeloma. Studies have shown that general anesthesia has a higher risk of complications over regional anesthesia. Reduced sympathetic tone in regional anesthesia results in enhanced venous blood flow and the prevention of venous stagnation, which lowers the incidence of venous thromboembolism and fatal pulmonary embolism.^7^ Transfusion requirement, pneumonia, respiratory depression, myocardial infarct and renal failure are decreased significantly in regional anesthesia compared to general anesthesia.^8^ Incidence of delirium, postoperative need for ICU admission, length of hospital stay and need of ventilator care was found to be significantly reduced with regional anesthesia.^9^, ^10^ Regional anesthesia is currently an important aspect of multimodal enhanced recovery after surgery (ERAS) protocols, which seek to lower costs, increase safety, and improve patients' subjective experiences both during and after hospitalization.^14^ Since MM patients are already predisposed to a wide range of complications that could further be worsened by general anesthesia, PENG block followed by spinal block was the preferred anesthetic in this case.

Peripheral nerve blocks like PENG block have a low impact on hemodynamics and cardiovascular performance and are likely the best option for high‐risk patients who are intolerant to even minor hemodynamic changes.^5^, ^15^ PENG block refers to a new regional block technique that blocks the femoral nerve, obturator nerve, and accessory obturator branches that supply the anterior hip capsule. It can deliver ideal analgesia without compromising the patient's muscle strength, promoting the postoperative functional recovery of the patient. Supine position is a significant advantage of the PENG block, which is especially important for individuals with chronic pain or acute hip fractures.^16^ PENG block allows having a pain‐free positioning for spinal anesthesia procedure, with relatively no motor weakness and prolonged analgesic efficacy.^6^

Literature has shown minimized risk when spinal, interscalene and superficial cervical blocks were performed in a 61 year multiple myeloma patient with tibial and humeral pathological fractures instead of general anesthesia.^17^ In another literature, psoas compartment block and an ultrasound guided sciatic block was done for the anesthetic management of a 44 year patient with a fractured femur, accompanied by diagnoses of MM and acute renal failure, and the literature suggested that it was a good alternative to other forms of anesthesia to decrease the rate of complications.^18^

In this case, general anesthesia was avoided so as to avoid its complications. After an intravenous bolus dose of fentanyl, PENG block was given to obliterate pain while positioning the patient for spinal anesthesia as hip fracture is an extremely painful condition, especially in a patient with multiple myeloma. The rest of the fracture fixation was conducted under spinal anesthesia and postoperatively, pain was managed with dexmedetomidine and paracetamol infusions.

The patient had no intraoperative or postoperative complications and showed significant progress during his follow up visits. With carefully planned preoperative measures, proper anesthetic techniques and close monitoring of the patient after the surgery, possible complications were averted.

## 5 CONCLUSION

To our knowledge, this is the first case report regarding use of PENG block followed by regional anesthesia for management of pathological fracture in multiple myleoma patient in Nepal. In order to guarantee optimal outcomes, a comprehensive preoperative evaluation should be carried out for significant comorbidities prior to the surgery. Proper anesthetic techniques should be employed to minimize both intraoperative and postoperative complications. Regional anesthesia can be used to provide effective anesthesia in such patients. Proper monitoring of the patient postoperatively is crucial. Controlling pain requires a multimodal approach. An effective anesthetic plan and communication between surgeons, anesthesiologists and hematologists can ensure the best possible outcome for patients.

## AUTHOR CONTRIBUTIONS

**Aayusha Suwal:** Conceptualization; data curation; investigation; resources; supervision; writing – original draft. **Nischal Subedi:** Data curation; investigation; project administration; resources; writing – original draft. **Nischal Shrestha:** Conceptualization; data curation; investigation; resources; supervision. **Ujma Shrestha:** Investigation; resources; supervision.

## FUNDING INFORMATION

None.

## CONFLICT OF INTEREST STATEMENT

The authors declare no conflict of interest.

## CONSENT

Written informed consent was obtained from the patient to publish this report in accordance with the journal's patient consent policy.

## Data Availability

No data were used.
